# Antileishmanial Activity, Toxicity and Mechanism of Action of Complexes of Sodium Usnate with Lanthanide Ions: Eu(III), Sm(III), Gd(III), Nd(III), La(III) and Tb(III)

**DOI:** 10.3390/ijms25010413

**Published:** 2023-12-28

**Authors:** Fernanda da Silva, Yasmin Silva Rizk, Amarith Rodrigues das Neves, Estela Mariana Guimarães Lourenço, Alda Maria Teixeira Ferreira, Melquisedeque Mateus Monteiro, Dênis Pires de Lima, Renata Trentin Perdomo, Iluska Senna Bonfá, Mônica Cristina Toffoli-Kadri, Adriana Pereira Duarte, Daniel Mendes Nunes, Marco Antonio Utrera Martines, Eliane Mattos Piranda, Carla Cardozo Pinto de Arruda

**Affiliations:** 1 Laboratório de Parasitologia Humana, Instituto de Biociências, Universidade Federal de Mato Grosso do Sul (UFMS), Campo Grande 79070-900, Brazil; s.fernanda@ufms.br (F.d.S.); yasminrizk@gmail.com (Y.S.R.); amardasneves@gmail.com (A.R.d.N.); eliane.piranda@ufms.br (E.M.P.); 2 Laboratório de Síntese e Transformação de Moléculas Orgânicas-SINTMOL, Instituto de Química, Universidade Federal de Mato Grosso do Sul (UFMS), Campo Grande 79074-460, Brazil; estela.mariana@hotmail.com (E.M.G.L.); denis.lima@ufms.br (D.P.d.L.); 3 Laboratório de Imunologia, Biologia Molecular e Bioensaios, Instituto de Biociências, Universidade Federal de Mato Grosso do Sul (UFMS), Campo Grande 79070-900, Brazil; alda.ferreira@ufms.br; 4 Laboratório de Biologia Molecular e Culturas Celulares, Instituto de Biociências, Universidade Federal de Mato Grosso do Sul (UFMS), Campo Grande 79070-900, Brazil; melquisedequemateus@gmail.com (M.M.M.); renata.trentin@ufms.br (R.T.P.); 5 Laboratório de Farmacologia e Inflamação, Faculdade de Ciências Farmacêuticas, Alimentos e Nutrição, Universidade Federal de Mato Grosso do Sul (UFMS), Campo Grande 79074-460, Brazil; iluska.senna@ufms.br (I.S.B.); monica.kadri@ufms.br (M.C.T.-K.); 6 Instituto de Química, Universidade Federal de Mato Grosso do Sul (UFMS), Campo Grande 79074-460, Brazil; adriana.duarte@ufms.br (A.P.D.); marco.martines@ufms.br (M.A.U.M.); 7 Faculdade de Química, Universidade Estadual de Mato Grosso do Sul (UEMS), Campo Grande 79804-970, Brazil; danims@uems.br

**Keywords:** lanthanide complexes, biological activity, *Leishmania amazonensis*, cytotoxicity, selectivity, mechanism of action

## Abstract

Leishmaniases are neglected diseases with limited therapeutic options. Diffuse cutaneous leishmaniasis can occur in Brazil due to *Leishmania amazonensis*. This study details the antileishmanial activity and cytotoxicity of complexes of sodium usnate (SAU) with lanthanide ions ([LnL_3_ (H_2_O)_x_] (Ln = La(III), Nd(III), Gd(III), Tb(III), Eu(III) and Sm(III); L = SAU). All lanthanide complexes were highly active and more potent than SAU against *L. amazonensis* promastigotes and intracellular amastigotes (Pro: IC_50_ < 1.50 μM; Ama: IC_50_ < 7.52 μM). EuL_3_·3H_2_O and NdL_3_·3H_2_O were the most selective and effective on intracellular amastigotes, with a selectivity index of approximately 7.0. In silico predictions showed no evidence of mutagenicity, tumorigenicity or irritation for all complexes. Treatment with EuL_3_·3H_2_O triggered NO release even at the lowest concentration, indicating NO production as a mechanism of action against the parasite. Incubating promastigotes with the lanthanide complexes, particularly with SmL_3_·4H_2_O and GdL_3_·3H_2_O, led to a change in the mitochondrial membrane potential, indicating the ability of these complexes to target this essential organelle. The same complexes caused cell death through cell membrane disruption, but their relationship with early or late apoptotic processes remains unclear. Thus, the inclusion of lanthanide ions in SAU improves selectivity with a promising mechanism of action targeting the mitochondria.

## 1. Introduction

The World Health Organization (WHO) defines neglected tropical diseases (NTDs) as a group of diseases that are prevalent in the poorest regions of the world, where access to healthcare, sanitation and safe drinking water is inadequate [1]. During the COVID-19 pandemic, there has been a rise in mortality rates from NTDs due to insufficient support for affected individuals, who are often among the most vulnerable populations [2]. This reinforces what the Drugs for Neglected Diseases *initiative* (DNDi) has confirmed: there are no neglected diseases, only *neglected patients* [3].

Leishmaniases are among the top ten NTDs. These are a group of vector-borne infectious diseases that affect more than 12 million people worldwide, with 0.9–1.6 million new cases each year [4]. The potentially fatal visceral manifestation or even the potentially disfiguring and stigmatizing cutaneous and/or mucocutaneous forms are caused by *Leishmania* protozoan parasites. This broad spectrum of clinical manifestations is related to the variety of parasite species: more than 20 species can cause the disease, and these are transmitted by a diversity of phlebotomine species in various epidemiological cycles [5]. 

Cutaneous leishmaniasis (CL) produces skin lesions with a tendency to ulceration and can evolve into destructive lesions on the mucous membranes (mucosal or mucocutaneous leishmaniasis, ML). Of the cases, 95% occur in the Americas, the Mediterranean basin, the Middle East and Central Asia [4]. *Leishmania* (*Leishmania*) *amazonensis* Lainson and Shaw, 1972 is one of the pathogens responsible for cutaneous leishmaniasis (CL) in Brazil. It causes either localized lesions or the diffuse form of the disease, in which the parasite spreads due to an impaired cell-mediated immune response [6]. Consequently, lesions tend to be more resistant to treatment and do not heal spontaneously [7].

The antileishmanial drugs currently used include pentavalent antimonials (N-methyl glucamine antimonate and sodium stibogluconate), amphotericin B (deoxycholate and the less toxic liposomal formulation), the antibiotic pentamidine and miltefosine, an oral anticancer drug currently used to treat cutaneous and visceral leishmaniasis [8]. In addition, controlled released drug systems carried out by nanoparticles became an important tool to improve antileishmanial chemotherapy [9,10,11]. Therapeutic options for leishmaniases are limited, toxic and costly, in addition to being poorly accessible to patients due to the repetitive parenteral route of administration [8]. Thus, the development of safer and shorter treatments is highly desirable. Mitochondria are considered potential targets for the development of antileishmanial drug candidates, as they are important organelles with unique properties and proteins that differ from those of the mammalian host [12]. As an example, paromomycin (paromomycin sulfate, C_23_H_47_O_18_S) is an intramuscularly administered aminoglycoside antibiotic for the treatment of leishmaniasis, whose mechanism of action involves mitochondria [13].

**Figure 1:**
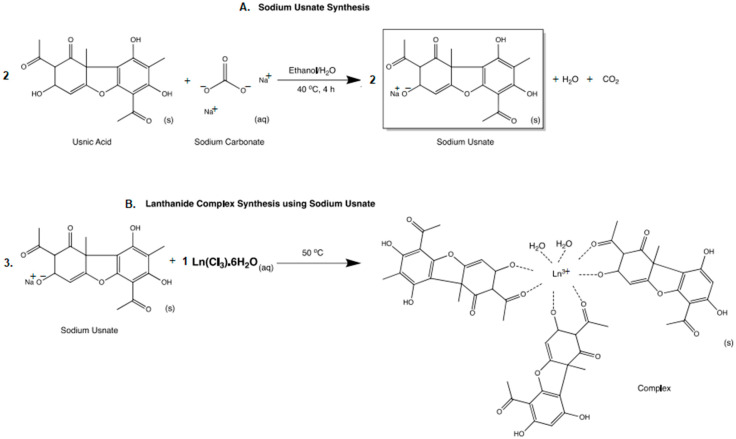
(**A**). Structural formula of usnic acid (R= OH) and sodium usnate **1** (SAU, R = ONa) [12]. (**B**). Complexes [Ln L_3_ (H_2_O)_x_] (Ln = Sm(III) **2**, Gd(III) **3**, Eu(III) **4**, Nd(III) **5**, Tb(III) **6**, La(III) **7** and L = sodium usnate) [21].

Lichens are organisms formed by the interspecific association of a photobiont (algae or cyanobacteria) and a mycobiont (fungus). This harmonious relationship results in more than 630 secondary metabolites: aliphatic acids, meta- and paradepsides, depsidons, benzyl esters, dibenzofurans, xanthones, anthraquinones, terpenes and derivatives of pulvinic acid [14]. However, the lichenic compound most studied is usnic acid (2,6-diacetyl-7,9-dihydroxy-8,9-dimethyl-1,3(2H,9bH)-dibenzofurandione (Figure 1), a dibenzofuran derivative with several biological activities, including antileishmanial [15,16,17]. 

The biological activities of usnic acid have been widely described, among which we can highlight the antibacterial, antiprotozoal, antiviral, anti-inflammatory, antipyretic and antitumor activities [16,18]. To enhance these activities, several changes have been made in the structure of the molecule, as its different functional groups make it a good target for structural modification [19]. Derivatization was also performed to overcome the low solubility of usnic acid and optimize the production of the compounds, as the acquisition of natural lichen biomass to supply secondary metabolites on a large scale is neither practical nor environmentally friendly [20].

Derici et al. [18] have shown that usnic acid has an apoptotic effect on several species of *Leishmania*. In order to increase the solubility of this compound in water, sodium usnate (SAU) **1** with the general formula NaL_2_·5H_2_O (Figure 1) was synthesized and complexed with metals to improve the activity [21]. The coordination compounds containing lanthanide ions that we present in this study have been obtained from the molecular modification of SAU **1**. Because of their high chemical reactivity, lanthanides never occur as pure elements in nature but only as sparsely distributed compounds that form rare minerals. Lanthanide compounds often have magnetic, catalytic and optical properties, and therefore they are currently widely used in industry and medicine as chemical markers and optical sensors [22].

In this work, the lanthanides samarium-Sm(III), gadolinium-Gd(III), europium-Eu(III), neodymium-Nd(III), lanthanum-La(III) and terbium-Tb(III) were complexed with SAU **1** [21], resulting in complexes that were tested for antileishmanial activity and selectivity. In addition to elucidating their mechanisms of leishmanicidal action, these promising results may contribute to more viable and effective therapeutic options against cutaneous leishmaniasis.

## 2. Results and Discussion

### 2.1. Antileishmanial Activity

Sodium usnate (SAU **1**) was synthesized to increase the solubility of usnic acid in water (Figure 1), looking for better suitability for biological in vitro and in vivo assays.

Biological applications of lanthanides have recently increased because they are abundant in the environment and exhibit chemistry similar or superior to other biologically useful metals because of their higher Lewis acidity. They have sufficient coordination chemistry to allow selective uptake, transport and incorporation into enzymes. Their biological coordination chemistry is analogous to that of other metals, most notably Ca^II^ and Fe^III^, but includes cooperative metal bonding to amplify the effects of small differences in ionic radius and allow selectivity [23,24,25,26,27,28].

In this work, SAU **1** and the lanthanide complexes **2**–**7** were tested against *L. (L.) amazonensis* promastigote forms, and cell viability was assessed by MTT metabolization, using the principle of tetrazolium–formazan conversion by living cell mitochondrial enzymes. All complexes were found to be highly active against promastigote forms (Table 1), more potent than the original compound SAU **1** (IC_50_ = 2.09 μM) and even more effective than the reference drug, pentamidine (IC_50_ = 1.50 μM). EuL_3_·3H_2_O **4** was more potent than the reference drug amphotericin B (IC_50_ = 0.20 versus 0.145 μM). The complex with Tb(III) 6 showed impressive activity (IC_50_ = 0.0023 μM), 63 times higher than amphotericin B and almost a thousand times higher than SAU **1** (Table 1). The optimization of the cytotoxic effect of the same lanthanide complexes over SAU was observed by Nunes et al. on MCF-7 tumor cells [21]. 

Derici et al. [18] observed a potent activity of usnic acid on promastigotes of *Leishmania major* Yakimoff and Schokhor, 1914, *L. (L.) infantum* Nicolle, 1908 and *L. tropica* Wright, 1903, with IC_50_ values/48 h of 10.76 µg·mL^−1^, 13.34 µg·mL^−1^ and 21.06 µg·mL^−1^, respectively. Similarly, Luz et al. [16] found an IC_50_/72 h = 18.30 µg·mL^−1^ for *L. (L.) infantum* promastigotes treated with usnic acid. In our work, sodium usnate SAU **1** was found to be highly active against *L. (L.) amazonensis* promastigotes, with an IC_50_/72 h = 2.09 µM (=0.77 µg·mL^−1^), showing that improved solubility in aqueous medium may have resulted in optimized antileishmanial activity.

All compounds were found to be more active against promastigotes than against amastigote forms. To understand this pharmacological behavior, it is important to emphasize that amastigotes are the intracellular stage of *Leishmania* parasites. Therefore, a drug candidate must be able to cross the macrophage cell membrane to reach these forms. In this context, biopharmaceutical aspects are crucial for predicting the biological tendencies of a molecule. The topological polar surface area (TPSA) is an important biopharmaceutical parameter considering molecular complexes and is directly related to the number of polar groups exposed to solvents. Complexes and peptoids with low TPSA values are already known to have a higher permeability ratio [29]. All complexes tested showed moderate TPSA values (Table 2). Considering that the in vitro activity on intracellular amastigotes was demonstrated in RPMI 1640 medium, the intermolecular interaction between the polar groups of the complexes and the aqueous medium may have reduced their permeability across the membrane, which explains the inferior results.

In addition, biopharmaceutical parameters are useful to indicate whether a compound may have oral viability. This route of administration is desirable for the treatment of most diseases, such as leishmaniasis. Advantages include ease of administration, cost-effective manufacturing, easy storage conditions, high patient compliance and more accurate self-administered dose [30]. In particular, the logP value has been widely discussed in the postulation of the rule of five and is still considered an important parameter of a prototype. This parameter can be used to predict whether a compound will penetrate the cell membrane or be easily soluble in an aqueous medium such as blood [31]. According to Lipinski et al. [32], a logP ≤ 5 is considered necessary for an ideal prototype. Meanwhile, logS values are directly related to the water solubility of a compound, and values close to −4 have been defined as the standard for a drug candidate [33]. All tested compounds exhibited logS values close to −4 and logP ≤ 5, following the postulated characteristics and indicating that the complexes can be administered orally (Table 2).

Intracellular amastigote activity was evaluated according to the following arbitrary scale: compounds were considered active when IC_50_ < 20 µM, moderately active when 20 < IC_50_ < 50 µM, and potentially inactive when IC_50_ > 50 µM [34]. Therefore, all lanthanide complexes were active against *L. (L.) amazonensis* amastigotes (Table 1), with a greater effect than SAU **1**. Two-way analysis of variance (ANOVA) followed by the Tukey test has confirmed that NdL_3_·3H_2_O **5** was statistically different from SAU **1** (*p* ≤ 0.0001) at all concentrations tested. EuL_3_·3H_2_O **4** was more potent than SAU at the lowest concentrations (*p* ≤ 0.0001) and TbL_3_·2H_2_O **6** only at the higher concentration (50 µg/mL; *p* ≤ 0.0001); all the other complexes were more potent than SAU **1** at all the concentrations tested excepting 6.25 µg/mL (*p* ≤ 0.0001).

These results lead to the conclusion that the insertion of the lanthanide ion led to the optimization of the antileishmanial activity. Because of their high chemical reactivity, it is not possible to analyze the activity of isolated lanthanides. This is the case with other drugs in medicine, such as the use of cyclodextrin to promote the stability of gold nanoparticles and the programmed release of antitumor drugs [35,36]. For example, the stability of trivalent antimonials (SbIII) is possible when linked to meglumine and gluconate, as in the antileishmanial drugs Glucantime^®^ and Pentostam^®^ [37].

**Figure 2:**
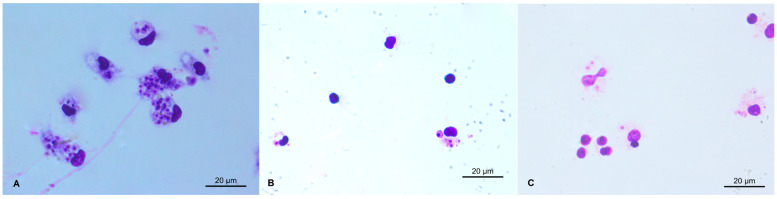
In vitro antileishmanial activity on *Leishmania (Leishmania) amazonensis* intracellular amastigotes. (**A**). Nontreated infected peritoneal cells. (**B**). Cells treated with NdL_3_·3H_2_O at a concentration of 6.5 μg·mL^−1^. (**C**). Cells treated with EuL_3_·3H_2_O at 12.5 μg·mL^−1^.

Among the complexes tested, those with Eu(III) **4** and Nd(III) **5** were the most active in intracellular amastigotes (IC_50_ = 2.98 and 2.83 μM, respectively; Figure 2), while the compound with Tb(III) was the least active (IC_50_ = 7.52 μM). Studies with other lanthanide complexes on other parasite species have shown similar results. Caballero et al. [38] observed that the complexes with Eu(III) and Nd(III) had the same effect as the reference drug Glucantime^®^; on the other hand, the complex with La(III) was the most active on *L. (L.) infantum* and on *L. (L.) braziliensis* amastigotes. In the present study, LaL_3_·3H_2_O **7** was active on *L. (L.) amazonensis* (IC_50_ = 4.58 μM), opening a promising perspective for application against several species of *Leishmania*. 

### 2.2. Cytotoxicity and Selectivity Index in Mammalian Cells

In silico predictions were used to estimate the risk of mutagenicity, tumorigenicity and irritation, and the complexes showed no evidence of these side effects (Table 2). To demonstrate host cell safety, SAU **1** and lanthanide complexes **2**–**7** were tested on NIH/3T3 fibroblasts to assess their cytotoxicity and estimate their selectivity, as recommended by the Organisation for Economic Co-operation and Development (OECD) [39].

A compound is considered to have low cytotoxicity when SI ≥ 10 [40]. The most active complexes on intracellular amastigotes were EuL_3_·3H_2_O **4** (IC_50_ = 2.98 µM; SI = 6.73) and NdL_3_·3H_2_O **5** (IC_50_ = 2.83 µM; SI = 6.97). Although they did not meet the SI ≥ 10 criteria, both showed a potent effect on *L. (L.) amazonensis* amastigotes. Súarez et al. obtained similar results [41] working with synthetic analogs of quinolones and alkaloids (1,2,3,4-tetra-hidro(benzo)-3-quinolin-ol and 2-amino-8-hidroxiquinoline), with SI~5.0. Indeed, the application of the SI criterion is controversial for in vitro tests used in drug research for infectious diseases [40]. Once in in vivo tests, these compounds induced a total reduction of lesions and no cytotoxicity-related clinical effects [41].

### 2.3. Nitric Oxide (NO) Production

The induction of a strong oxidative response by lanthanides on human cells has already been described, with the release of reactive oxygen species (ROS) that interfere with the signaling that regulates the immune response to an infection [24]. Macrophages are the host cells of *Leishmania* sp. and also play a role in the action against the parasite. The beneficial function of the ROS oxidative burst and NO is associated with the eradication of the parasite, in addition to the clinical improvement of the patient [42]. 

Intracellular parasites rely on the ability to undergo replication cycles within host cells. Once the cellular environment of macrophages becomes inviable, the parasite cannot replicate. NO production is one of the main mechanisms for the intracellular killing of the parasite. In addition to more immediate effects, such as reduced parasite metabolism and proliferation, it is associated with immune regulation of infection [43,44]. Paradoxically, NO alone may not be sufficient to control the infection and may contribute to the tissue damage observed in human CL [42].

**Figure 3:**
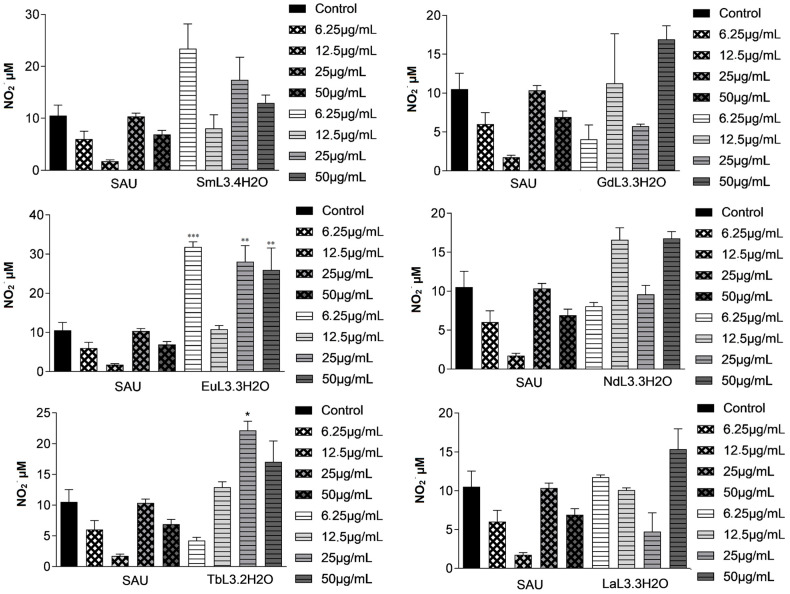
Release of nitric oxide by *L. (L.) amazonensis*-infected macrophages 24 h after treatment with sodium usnate (SAU **1**) and lanthanide complexes **2**–**7**. Bars represent the mean ± standard error of the mean (SEM) of three replicates. * *p* ≤ 0.05, ** *p* ≤ 0.01 and *** *p* ≤ 0.0001, for the different concentrations compared to untreated cells (control); ANOVA, followed by the Bonferroni test.

Treatment with lanthanide complexes at some concentrations induced an increase in NO release from infected macrophages (Figure 3). EuL_3_·3H_2_O **4** had the strongest effect at the very lowest concentration (6.25 µg·mL^−1^). This concentration is close to the half-maximal inhibitory concentration on the intracellular amastigotes (IC_50_ = 2.98 µM or 3.687 µg·mL^−1^), suggesting NO production as a mechanism of action of this complex against *L. (L.) amazonensis*. 

### 2.4. Flow Cytometric Analysis for Detection of Changes in Mitochondrial Membrane Potential (ΔΨm)

**Figure 4:**
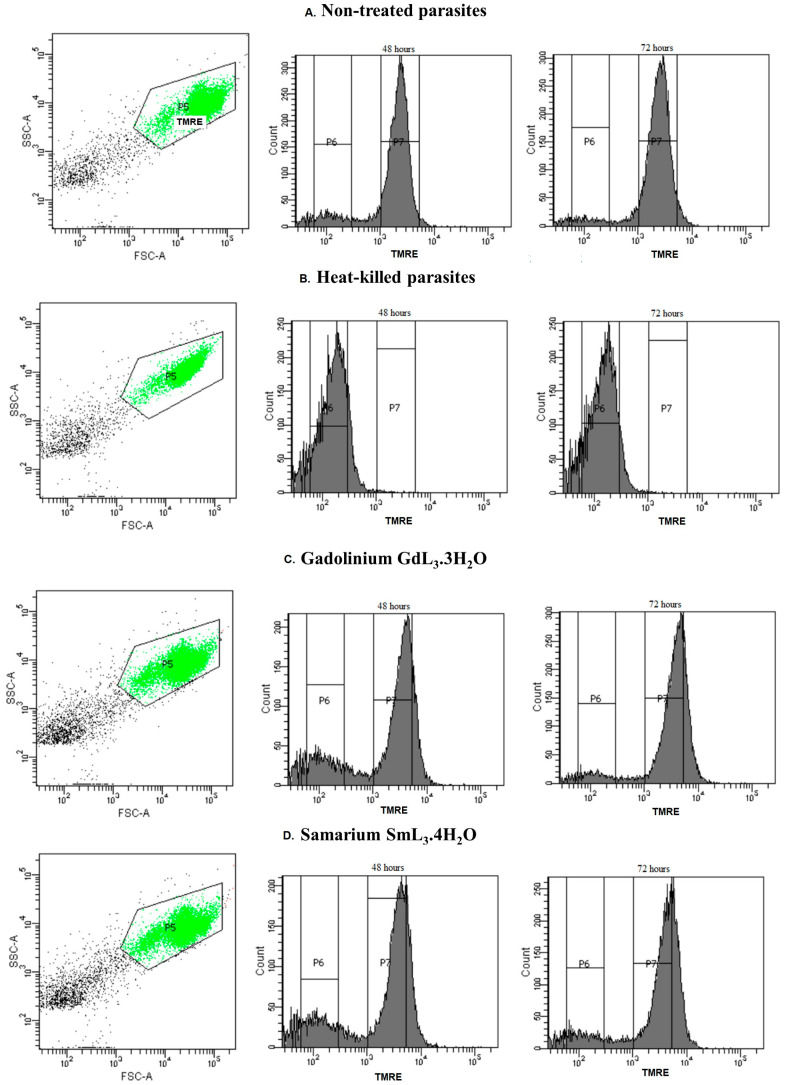
Flow cytometry of *L. (L.) amazonensis* to evaluate the mitochondrial membrane potential (ΔΨ_m_) after 48 and 72 h of treatment with lanthanide complexes [Ln L_3_ (H_2_O)_x_] (Ln= Gd(III) and Sm(III) and L= sodium usnate). Promastigotes captured in the gated region (dotplots) and representative histograms (% labeled cells) of promastigotes incubated with TMRE. (**A**) Non-treated promastigotes. (**B**) Heat-killed parasites. (**C**) Promastigotes treated with GdL_3_·3H_2_O **3** (1.50 μM). (**D**) Promastigotes treated with SmL_3_·4H_2_O **2** (1.26 μM).

Promastigotes treated with SAU **1** and lanthanide complexes **2**–**7** were stained with TMRE to evaluate changes in mitochondrial membrane potential (ΔΨm). Figure 4 and Table 3 show the histograms (%) of TMRE-labeled promastigotes after incubation with **1**–**7** at the calculated IC_50_ (Table 1). The complex with Sm(III) showed a marked effect on the mitochondrial membrane potential, as we observed a reduction in labeling of 40.7% and 41.7% after 48 and 72 h of treatment, respectively. Similarly, the complex with Gd(III) was able to reduce the ΔΨm by 36.2% and 24.5% after 48 and 72 h treatment, respectively. Complexes with La(III) and Nd(III) affected the ΔΨm in 22.3% and 11.35%, respectively, after 72 h of treatment. Thus, we can assume that the complexes with these lanthanides cause mitochondrial dysfunction, an important and recognized target of antileishmanial drugs.

SAU **1** had a mild effect on ΔΨm 72 h after treatment, with a 12% reduction in labeling at the concentration tested (IC_50_ = 2.09 μM). There are few reports on the effect of usnic acid on trypanosomatid mitochondria. Carvalho et al. [45] showed damage to the mitochondria of *Trypanosoma cruzi* epimastigotes treated with 30–50 μg·mL^−1^ usnic acid, with marked swelling and branching, as well as loss of organization of the mitochondrial cristae observed in ultrastructural analysis. Similarly, Luz et al. [16] observed changes in the mitochondrial morphology of *L. (L.) infantum* promastigotes treated with 25 μg·mL^−1^ usnic acid, with marked swelling. 

### 2.5. Flow Cytometric Analysis for Detection of Apoptosis and Membrane Permeability

*L. (L.) amazonensis* promastigote forms were treated with SAU **1** and the lanthanide complexes **2**–**7** at the calculated IC_50_ (Table 1) for 24, 48 and 72 h and then stained with PE Annexin V and 7-AAD for FACS analysis. This measurement can track cells over time from PE Annexin V and 7-AAD negative (viable or without measurable apoptosis) to PE Annexin V and 7-AAD positive (early apoptosis, membrane integrity present) and finally to PE Annexin V and 7-AAD positive (terminal apoptosis and death). The movement of parasites through these three stages is indicative of apoptosis. On the other hand, cells that are both PE Annexin V and 7-AAD positive may have undergone apoptotic death or died by a necrotic pathway. 

**Figure 5:**
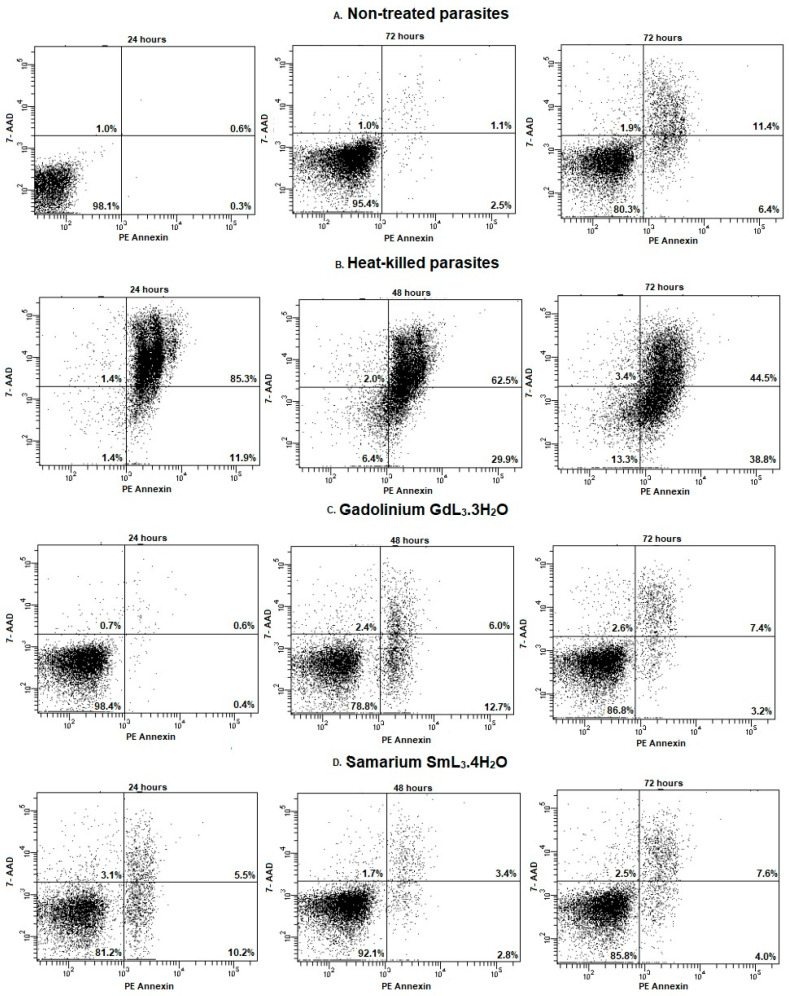
Flow cytometry of *L. (L.) amazonensis* to detect apoptosis or plasma membrane permeability after 24, 48 and 72 h of treatment with lanthanide complexes [Ln L_3_ (H_2_O)_x_] (Ln= Gd(III) and Sm(III) and L= sodium usnate). Promastigotes captured in the gated region and representative histograms of promastigotes incubated with PE Annexin V and 7-AAD. (**A**) Non-treated promastigotes. (**B**) Heat-killed parasites. (**C**) Promastigotes treated with GdL_3_·3H_2_O **3** (1.50 μM). (**D**) Promastigotes treated with SmL_3_·4H_2_O **2** (1.26 μM).

Figure 5 and Table 3 show the percentage of cell labeling after incubation with PE annexin and 7-AAD. Again, the effect of the complexes with the lanthanides Sm(III) and Gd(III) on the parasites can be highlighted. SmL_3_·4H2O **2** caused an earlier cell death effect since 24 h after treatment, there was a 17.2% reduction in viability, with 10.2% 7-AAD staining and 5.5% double labeling. On the other hand, GdL_3_·3H2O **3** had its greatest effect 48 h after treatment, also reducing cell viability by 17.4%, with 12.7% 7-AAD staining and 6% double labeling. Despite the disruption of cell membrane integrity in both cases, it was not possible to establish a relationship with either early or late apoptotic processes, as PE Annexin V labeling was low at all times studied.

### 2.6. Study Limitations

The complexity of understanding the mechanisms of leishmanicidal action, namely the induction of apoptosis, may be related to the use of IC_50_ as the inhibitory concentration for promastigote forms. The use of IC_90_ or IC_99_, or higher concentrations, would improve these analyses by promoting complete parasitic inhibition. Likewise, investigating the mechanisms of action of the complexes on the intracellular amastigote forms could greatly enhance our understanding despite the current methodology’s challenging nature.

## 3. Materials and Methods

### 3.1. Lanthanide Complexes

Compounds **1**–**7** (Figure 1) were synthesized in the Institute of Chemistry of the Federal University of Mato Grosso do Sul [19]. Sodium usnate (SAU) **1** was prepared as described in the literature [46] from the reaction of the ethanolic suspension of usnic acid (3 mmol) with a sodium carbonate solution (1.5 mmol) with stirring at 40 °C for 4 h, followed by filtration and evaporation of the solvent to obtain a yellow solid. Lanthanide complexes were then synthesized and characterized [21]. Briefly, sodium usnate (0.3 mmol) was dissolved in water and ethanol (3:1); 0.1 mmol of lanthanide ions: Sm(III), Gd(III), Eu(III), Nd(III), Tb(III) and La(III) were added to this solution and kept under stirring for 8 h at 50 °C. The resulting yellow precipitate was washed with ice water and dried in a vacuum desiccator (Figure 1). The general coordination formula was used: [LnL_3_(H_2_O)_x_], where L= C_18_H_15_O_7_, Ln = Tb(III), x = 2; Ln = Gd(III), Eu(III), Nd(III), La(III), x = 3 and Ln = Sm(III), x = 4.

### 3.2. Parasites

A standard strain of *Leishmania (Leishmania) amazonensis* (IFLA/BR/1967/PH8) was used for in vitro antileishmanial activity assays. Promastigote forms were grown in Schneider’s insect medium (Sigma-Aldrich^®^, São Paulo, Brazil) supplemented with 20% fetal bovine serum (Sigma-Aldrich^®^, São Paulo, Brazil), 10,000 U·mL^−1^ penicillin and 10 mg·mL^−1^ streptomycin (Sigma-Aldrich^®^, São Paulo, Brazil). Parasites were routinely isolated from skin lesions previously induced in BALB/c mice and maintained in axenic culture until passage 20 [34].

### 3.3. Animals

Four-week-old female BALB/c mice were obtained from the Central Animal Facility of the Federal University of Mato Grosso do Sul (UFMS). The animals were housed in mini-isolators connected to a ventilated rack, fed a balanced diet (Nuvital^®^, Nuvilab^®^, Seoul, Republic of Korea) and given ad libitum access to filtered water. This study received approval from the local Animal Experimentation Ethical Committee (CEUA/UFMS) under protocol 1,172/2021.

### 3.4. In Vitro Antileishmanial Activity on L. (L.) amazonensis Promastigote Forms

Promastigote forms of *L. (L.) amazonensis* in the logarithmic growth phase (10^6^ parasites/mL) were added to 96-well plates and incubated with SAU **1** and the lanthanide complexes **2**–**7** (0.78–50.0 µg·mL^−1^) in six replicates. The microplates were incubated at 26 °C for 72 h. Cell viability was assessed by adding 1 mg·mL^−1^/well of MTT ([3-(4,5-dimethylthiazol-2-yl)-2,5-diphenyltetrazolium bromide] (Sigma-Aldrich^®^ São Paulo, Brazil) [47]. Results were expressed as the half-maximal inhibitory concentration (IC_50_) calculated from a non-linear dose–response regression curve using the GraphPad PRISM 5.0 statistical program. Pentamidine (Sigma-Aldrich ^®^ São Paulo, Brazil; 0.19–12.5 µg·mL^−1^) and AMB (Amphotericin B, Sigma-Aldrich ^®^ São Paulo, Brazil; 0.15–1 µg·mL^−1^) were used as positive controls and pure Schneider^®^ culture medium as negative control.

### 3.5. In Vitro Antileishmanial Activity on L. (L.) amazonensis Intracellular Amastigotes

Peritoneal macrophages were obtained from BALB/c mice in RPMI 1640 culture medium (Sigma-Aldrich^®^ São Paulo, Brazil). After exclusion of inviable cells with trypan blue staining (Sigma-Aldrich^®^ São Paulo, Brazil), cells were transferred to 24-well plates with 13 mm round glass coverslips (10^6^ cells/well) and kept at 37 °C/5% CO_2_. After adhesion for one hour, cells were infected with promastigote forms of *L. (L.) amazonensis* in the stationary phase (4 × 10^6^ cells/well). After 4 h of incubation, samples (complexes **1**–**7**) were added in six replicates (6.25–50.0 µg·mL^−1^). Nontreated infected cells were used as the negative control, and AMB (0.125–1.0 µg·mL^−1^) and Pentamidine (6.25–50.0 µg·mL^−1^) were used as the positive controls. Coverslips were removed after 24 h, subjected to a dehydration battery with increasing concentrations of acetone: xylol and stained with Giemsa (Sigma-Aldrich^®^, São Paulo, Brazil) diluted 1:10 in distilled water [34]. After mounting on slides, 200 cells per coverslip were analyzed by light microscopy and the average number of amastigotes/cells was calculated. IC_50_ was determined using a non-linear dose–response regression curve as described above. The concentrations of complexes were compared by Two-way ANOVA followed by the Tukey test.

### 3.6. In Silico ADMET Predictions

All compounds were subjected to the OSIRIS Property Explorer program to estimate the risk of side effects such as mutagenicity, tumorigenicity and irritation. Furthermore, drug-related properties were measured, including clogP, logS, MW, drug similarity and overall drug score [33].

### 3.7. Cytotoxicity in Mammalian Cells

The cytotoxic effect of compounds **1**–**7** was evaluated on NIH/3T3 fibroblasts [48] obtained from the Rio de Janeiro Cell Bank (Duque de Caxias, Brazil). Fibroblasts were seeded at 5 × 10^5^ cells/well in 96-well plates. After 24 h of fixation, cells were incubated for 48 h with **1**–**7** at 2.5–250 µg·mL^−1^, in three replicates. Cell growth was evaluated using the sulforhodamine B colorimetric method [48], and the optical with absorbances read on the PT-READER microplate instrument (Spectramax 190, Molecular Devices, Sunnyvale, CA, USA) at 540 nm. DMSO (dimethyl sulfoxide, Sigma-Aldrich^®^ São Paulo, Brazil) was used as a negative control at a concentration necessary to solubilize the highest concentration of the test sample and did not interfere with cell viability. The growth inhibition percentage-GI (%) of each test sample was calculated in Microsoft Office Excel 2007 software [49]. IC_50_ was determined graphically in the Microcal Origin Version 6.00 program [50]. The selectivity index (SI) was calculated as the ratio between the cytotoxicity on cells (IC_50_) and the activity on the parasite forms (IC_50_).

### 3.8. NO Production by Infected Cells

Nitric oxide (NO) production by infected and treated peritoneal macrophages was estimated in the supernatants from the intracellular amastigotes assay by the Griess reaction [51]. Aliquots of the supernatants (50 mL) were incubated with Griess reagent (1:1) for 10 min at room temperature. Absorbance was read on a microplate reader (HumanReader HS, Wiesbaden, Germany) at 550 nm. Conversion of nitrite (NO^−2^) to micromolar (µM) was performed by comparing the samples to a standard curve obtained with known concentrations (1–10 µM) of sodium nitrite diluted in RPMI 1640 medium (Sigma-Aldrich^®^ São Paulo, Brazil). The average and standard error of the mean (SEM) were calculated from three replicates. The data were analyzed using ANOVA and subsequently subjected to the Bonferroni test.

### 3.9. Flow Cytometric Analysis to Detect Changes in Mitochondrial Membrane Potential (ΔΨm)

The mitochondrial membrane potential (∆Ψm) can change in cells undergoing apoptosis, oxidative stress, necrosis and other cellular processes. Cationic and lipophilic ∆Ψm-sensitive dyes accumulate within the mitochondria of healthy cells but not in mitochondria that have lost ∆Ψm. In this work, *L. (L.) amazonensis* promastigote forms (1 × 10^6^ mL^−1^) were treated for 48 and 72 h with SAU **1** and the lanthanide complexes **2**–**7** at the calculated IC_50_ (Table 1), in Schneider’s insect medium (Sigma-Aldrich^®^) supplemented with 20% FCS (Cultilab^®^, São Paulo, Brazil), 10,000 U·mL^−1^ penicillin and 10 mg·mL^−1^ streptomycin (Sigma-Aldrich^®^). Heat-killed parasites (60 °C bath/10 min) were used as positive control, and nontreated parasites were used as negative control [52]. Promastigotes were stained with 50 nM tetramethylrhodamine, ethylester (TMRE) (MitoStatus TMRE, BD Pharmingen^TM^, San Diego, CA, USA) for 15 min at room temperature and subjected to flow cytometric analysis (BD FACSCanto^TM^ II, Kingston, ON, Canada). Then, 10,000 events were acquired in the region previously determined to correspond to the parasites. Fluorescence was quantified and histograms were generated.

### 3.10. Flow Cytometry Analysis for Detection of Apoptosis 

PE Annexin V staining precedes the loss of membrane integrity observed in the final stages of cell death, resulting from either apoptotic or necrotic processes. PE Annexin V is used in conjunction with the vital dye 7-Amino-Actinomycin (7-AAD) to identify early apoptotic cells (7-AAD negative, PE Annexin V positive). Viable cells with intact membranes exclude 7-AAD, but the membranes of dead and damaged cells are permeable to 7-AAD. Cells that are in late apoptosis or already dead are both PE Annexin V and 7-AAD positive. In this work, *L. (L.) amazonensis* promastigote forms (1 × 10^6^ mL^−1^) were treated for 24, 48 and 72 h with **1**–**7** at the calculated IC_50_ (Table 1) in Schneider’s insect medium (Sigma-Aldrich^®^) supplemented with 20% FCS (Cultilab^®^), 10,000 U·mL^−1^ penicillin and 10 mg·mL^−1^ streptomycin (Sigma-Aldrich^®^). Heat-killed parasites (60 °C bath/10 min) were used as positive control, and untreated parasites were used as negative control [52]. Promastigotes were stained with 5 µL of PE Annexin V and 5 µL 7-AAD (PE Annexin V Apoptosis Detection Kit I, BD Biosciences^TM^, Franklin Lakes, NJ, USA) and immediately subjected to flow cytometric analysis (BD FACSCanto^TM^ II). Then, 10,000 events were captured in the region previously determined to correspond to the parasites. Fluorescence was quantified and histograms were generated.

## 4. Conclusions

All lanthanide complexes **2**–**7** were highly active and more potent than SAU **1** against both parasite forms (Pro: IC_50_ < 1.50 μM; Ama: IC_50_ < 7.52 μM). The most effective complexes on promastigotes were TbL_3_·2H_2_O **6** and EuL_3_·3H_2_O **4**. EuL_3_·3H_2_O **4** and NdL_3_·3H_2_O **5** were the most selective and effective on intracellular amastigotes, with an SI of approximately 7.0. Treatment with EuL_3_·3H_2_O **4** triggered NO release even at the lowest concentration (6.25 μg·mL^−1^), indicating NO production as a mechanism of action against *L. (L.) amazonensis*. Incubation of promastigotes with the lanthanide complexes at the calculated IC_50_, especially with SmL_3_·4H_2_O **2** and GdL_3_·3H_2_O **3**, was found to modulate the mitochondrial membrane potential, suggesting that these complexes are able to target this crucial organelle. The aforementioned complexes caused cell death through cell membrane disruption, but their relationship with early or late apoptotic processes remains unclear. Further research is being conducted to understand the mechanisms of leishmanicidal action at different concentrations.

## Figures and Tables

**Table 1:** In vitro antileishmanial activity, cytotoxic effect on mammalian cells and selectivity index of SAU **1** and lanthanide complexes **2**–**7**.

Lanthanide Complex	*L. amazonensis* Promastigotes (Pro.) IC_50_ ^1^ (μM)	*L. amazonensis* Intracellular Amastigotes (Ama.) IC_50_ ^2^ (μM)	Fibroblasts NIH/3T3 (Fib.) IC_50_ ^3^ (μM)	SI ^4^ (Ama.)
SAU (sodium usnate) **1**	2.09	19.97	68.20	3.41
SmL_3_·4H_2_O **2**	1.26	5.23	19.98	3.82
GdL_3_·3H_2_O **3**	1.50	5.52	26.54	4.81
EuL_3_·3H_2_O **4**	0.20	2.98	20.07	6.73
NdL_3_·3H_2_O **5**	0.91	2.83	19.73	6.97
TbL_3_·2H_2_O **6**	0.0023	7.52	17.49	2.32
LaL_3_·3H_2_O **7**	1.49	4.58	10.89	2.38
Pentamidine	1.49	0.92	NT	NC
Amphotericin B	0.145	0.198	NT	NC

^1^ IC_50_: half-maximal inhibitory concentration on promastigotes; ^2^ IC_50_: half-maximal inhibitory concentration on intracellular amastigotes; ^3^ IC_50_: half-maximal inhibitory concentration on NIH/3T3 fibroblasts; ^4^ SI (selectivity index): IC_50_ in NIH/3T3 fibroblasts/IC_50_ on intracellular amastigotes; positive controls: pentamidine and amphotericin B; NC: not calculated; NT: not tested. Data are representative of three independent experiments.

**Table 2:** In silico absorption, distribution, metabolism, excretion and toxicity (ADMET) predictions of lanthanide complexes. No mutagenic, tumorigenic or irritating effects were observed for any of the complexes.

Lanthanide Complex	logP	logS	TPSA	Drug Likeness	Drug-Score
SAU (sodium usnate) **1**	−1.31	−3.52	123.9	−0.34	0.36
SmL_3_·4H_2_O **2**	0.76	−3.52	121.1	−0.09	0.37
GdL_3_·3H_2_O **3**	0.76	−3.52	121.1	−0.09	0.37
EuL_3_·3H_2_O **4**	0.76	−3.52	121.1	−0.09	0.37
NdL_3_·3H_2_O **5**	0.76	−3.52	121.1	−0.09	0.37
TbL_3_·2H_2_O **6**	0.76	−3.52	121.1	−0.09	0.37
LaL_3_·3H_2_O **7**	0.76	−3.52	121.1	−0.09	0.37
Pentamidine	1.75	−2.23	118.2	−5.35	0.45
Amphotericin B	0.32	−5.08	319.6	−0.14	0.27

LogP = partition coefficient; LogS = water solubility; TPSA = topological polar surface area; Druglikeness = similarity of the properties between compounds and existing drugs; Drug-score = compilation of ADMET parameters to judge the compound’s overall potential to qualify for a drug.

**Table 3:** Flow cytometry of *L. (L.) amazonensis* to evaluate the mitochondrial membrane potential (ΔΨ_m_) and apoptosis after treatment with SAU **1** and lanthanide complexes **2**–**7**. Promastigotes captured in the gated region and representative histograms (% labeled cells) of promastigotes incubated with TMRE, PE Annexin V and 7-AAD.

Treatment	Time after Treatment TMRE		Time after Treatment PE Annexin V and 7-AAD											
	48 h	72 h	24 h				48 h				72 h			
			Unst.	An.	7-AAD	An./ 7-AAD	Unst.	An.	7-AAD	An./ 7-AAD	Unst.	An.	7-AAD	An./ 7-AAD
Nontreated parasites	73.3	78.4	98.1	1.0	0.3	0.6	95.4	1.0	2.5	1.1	80.3	1.9	6.4	11.4
Heat-killed parasites	0.4	0.2	1.4	1.4	11.5	85.3	6.4	2.0	29.0	62.5	13.3	3.4	38.8	44.5
SAU **1**	69.3	69.0	98.5	0.6	0.4	0.5	96.0	1.4	0.8	1.9	85.7	2.3	3.8	8.2
SmL_3_·4H_2_O **2**	43.5	45.7	81.2	3.1	10.2	5.5	92.1	1.7	2.8	3.4	85.8	2.5	4.0	7.6
GdL_3_·3H_2_O **3**	46.8	59.2	98.4	0.7	0.4	0.6	78.8	2.4	12.7	6.0	86.8	2.6	3.2	7.4
EuL_3_·3H_2_O **4**	79.9	80.6	98.2	0.6	0.5	0.7	95.4	1.3	1.0	2.3	84.0	1.9	6.0	8.1
NdL_3_·3H_2_O **5**	70.1	60.9	98.4	0.7	0.3	0.5	93.8	1.4	1.6	3.3	79.8	2.5	7.7	10.0
TbL_3_·2H_2_O **6**	70.2	69.5	98.3	0.8	0.2	0.6	94.5	1.3	1.2	3.0	80.9	2.6	6.4	10.1
LaL_3_·3H_2_O **7**	76.0	82.0	98.3	0.7	0.4	0.5	93.3	1.8	1.6	3.3	82.0	2.4	5.6	10.0

Unst. = unstained parasites; An. = PE Annexin V-stained parasites; 7-AAD = 7-AAD-stained parasites; An./7-AAD = double stained parasites.

## Data Availability

The data presented in this study are available on request from the corresponding author.
